# *Meloidogyne luci*: an emerging threat to agriculture and challenges in diagnostics and sustainable management

**DOI:** 10.3389/fpls.2026.1867596

**Published:** 2026-07-07

**Authors:** Jasmina Bačić, Ivana Lalićević, Nik Susič, Saša Širca, Barbara Gerič Stare, Maria L. Inácio, Carla Maleita

**Affiliations:** 1 Department of Plant Diseases, Institute for Plant Protection and Environment, Belgrade, Serbia; 2 Plant Protection Department, Agricultural Institute of Slovenia, Ljubljana, Slovenia; 3 Plant Health Department, National Institute for Agriculture and Veterinary Research, I.P., Oeiras, Portugal; 4 GREEN-IT Bioresources for Sustainability, Instituto de Tecnologia Química e Biológica António Xavier, Universidade NOVA de Lisboa, Oeiras, Portugal; 5 CERES - Chemical Engineering and Renewable Resources for Sustainability, University of Coimbra, Coimbra, Portugal

**Keywords:** integrated pest management, *Meloidogyne* spp., molecular identification, plant resistance, quarantine nematodes, *Meloidogyne luci*

## Abstract

The tropical root-knot nematode *Meloidogyne luci* is an obligate and highly polyphagous plant pathogen that infects root systems, inducing characteristic swellings (galls) which reduce both yield and crop quality in a wide range of agricultural plants, including vegetables, fruit crops, and ornamentals. The species was formally described in 2014 from populations collected in Brazil, Chile and Iran. *Meloidogyne luci* shares important morphological, biochemical, and molecular similarities with closely related species, making its accurate identification challenging. Since its description, the species has been reported in multiple European countries, raising concerns about its spread and establishment. Previously included in the European and Mediterranean Plant Protection Organization (EPPO) Alert List, *M. luci* was transferred to the EPPO A2 List in 2023 due to its potential agricultural impact within Europe. Accurate identification, based on a combination of morphological, biochemical, and molecular approaches, remains essential for reliable diagnosis and for supporting phytosanitary decision-making and management strategies. This overview summarizes current knowledge on the geographic distribution, host range, symptomatology, bio-ecology, diagnostic approaches, proposed control measures, and pest risk considerations for *M. luci*.

## 1 Introduction

Root-knot nematodes (RKN) of the genus *Meloidogyne* are one of the most widespread and polyphagous plant pathogens (Moens et al., 2009; Jones et al., 2013). These obligate sedentary endoparasites are among the most destructive plant parasitic nematodes of vegetables, and other crops (Tileubayeva et al., 2021). The plants infected with *Meloidogyne* species show above ground symptoms such as stunting, yellowing and chlorosis, while typically below ground symptoms are unusual swelling on roots called galls, which have negative effects on the uptake of water and nutrients resulting in yield reduction (Nicol et al., 2011). Additionally, mechanical wounding of the root improves the introduction of other pathogens, such as fungi, bacteria or virus leading to secondary infections (Bertrand et al., 2000).

So far, over 100 species have been described within *Meloidogyne* genus (Subbotin et al., 2021). The majority and most important studies have been focused on four RKN species considered as major pests in agriculture, especially in vegetable production: *M. incognita* (Kofoid & White, 1919) Chitwood, 1949; *M. javanica* (Treub, 1885) Chitwood, 1949; *M. arenaria* (Neal, 1889) Chitwood, 1949; and *M. hapla* Chitwood, 1949 (Karssen, 2002; Elling, 2013). Furthermore, several other RKN species like *M. chitwoodi* Golden, O’Bannon, Santo & Finley 1980; *M. enterolobii* Yang & Eisenback, 1983; *M. fallax* Karssen, 1996; *M. graminicola* Golden & Birchfield, 1965; *M. luci* Carneiro et al., 2014; and *M. mali* Itoh, Ohshima & Ichinohei, 1969 from A2 Quarantine List of the European Plant Protection Organization (EPPO), have become emerging and widespread pests in Europe (Wesemael et al., 2011; EPPO, 2023a). *Meloidogyne luci* is morphologically similar to the tropical species *M. ethiopica* Whitehead, 1968 and both can damage a wide range of economically important plants (EPPO, 2023b). *Meloidogyne ethiopica* was added to the EPPO Alert List in 2011, after being reported in European countries (Greece, Italy, and Slovenia) and Türkiye (Širca et al., 2004; Strajnar et al., 2009; Conceição et al., 2012; Maleita et al., 2012; Aydınlı et al., 2013) and before *M. luci* description, from different plants species in Brazil, Chile and Iran, by Carneiro et al. (2014).

Description of this new species and studies on RKN populations obtained from different geographical origins and host plants allowed the reclassification of all *M. ethiopica* European and Turkish populations identified up to date as *M. luci* (Gerič Stare et al., 2017; Janssen et al., 2016). Later, the analysis of morphological, biochemical and molecular characters of *M. luci* and *M. ethiopica* as well *M. inornata* Lordello, 1956, showed that these RKN species share high similarity and consequently, were classified together as *M. ethiopica* group (Carneiro et al., 2014; Gerič Stare et al., 2017, 2019). *Meloidogyne luci* was included in the EPPO Alert List in 2017, and a few years later, in 2023, was added to the EPPO A2 List (EPPO, 2023a). *Meloidogyne ethiopica* is still listed on EPPO A1 List while its first report on kiwifruit *Actinidia deliciosa* (A. Chev.) C.F. Liang & A.R. Ferguson in Türkiye, was only in 2024 (Felek and Akyazi, 2024).

Diagnosing *M. luci* is challenging due to its close morphological, biochemical and molecular similarity to *M. ethiopica* and *M. inornata*. Furthermore, this species poses a risk to agriculture because of its global distribution and wide host range. Use of conventional morphological, biochemical and molecular methods to identify *M. luci* is a crucial first step in its integrated management. Here, we review the current knowledge on geographic distribution, plant host range, symptoms of damage, bio-ecology, identification methods, proposed effective programs for *M. luci* control, and pest risk assessment considerations.

## 2 Geographic distribution, host range and symptoms

*Meloidogyne luci*, already recorded on four continents (Africa, South America, Asia and Europe (**Table 1**), is a polyphagous RKN species with a wide host range. Currently, 68 different plant species are identified as *M. luci* hosts (**Table 2**), and according to plant host range experiments, this species can multiply on many cultivated plants whereas only a few tested plant species are non-hosts (**Table 3**).

**Table 1:** Geographic distribution of *Meloidogyne luci* and respective host plants^1^.

Continent	Country	Host plants			References
		Family	Scientific name	Common name	
Africa	Ethiopia	Fabaceae	*Cicer arietinum* L.	Chickpea	Kefelegn et al., 2025
Asia	Iran	Rosacae	*Rosa* sp.	Rose	Carneiro et al., 2014
		Plantaginaceae	*Antirrhinum majus* L.	Snapdragon	Carneiro et al., 2014
		Crassulaceae	*Hylotelephium spectabile* (Boreau) H.Ohba	Sedum	Carneiro et al., 2014
	Türkiye	Actinidiaceae	*Actinidia chinensis* Planch.	Kiwi fruits	Aydınlı and Mennan, 2022
		Cucurbitaceae	*Cucumis sativus* L.	Cucumber	Aydınlı et al., 2013; Aydınlı and Mennan, 2016; Gerič Stare et al., 2017
		Fabaceae	*Phaseolus vulgaris* L.	Common bean	Aydınlı, 2018
		Solanaceae	*Capsicum annuum* L.	Pepper	Aydınlı, 2018
			*Solanum lycopersicum* L.	Tomato	Aydınlı et al., 2013; Aydınlı and Mennan, 2016; Gerič Stare et al., 2017; Aydınlı,2018
			*S. melongena* L.	Aubergine	Aydınlı and Mennan, 2016; Gerič Stare et al., 2017
Europe	Greece	Poaceae	*Zea mays* L.	Maize	Conceição et al., 2012; Gerič Stare et al., 2017
	Italy	Solanaceae	*Solanum lycopersicum* L.	Tomato	Maleita et al., 2012; Gerič Stare et al., 2017
	Portugal	Asparagaceae Oxalidaceae Solanaceae	*Cordyline australis* (G.Forst.) Endl. *Oxalis corniculata* L. *Solanum lycopersicum* L. *S. tuberosum* L.	Cabbage tree Creeping lady's sorrel Tomato Potato	Santos et al., 2019 Santos et al., 2019 Santos et al., 2019 Maleita et al., 2018; Rusinque et al., 2021
	Serbia	Cucurbitaceae Solanaceae	*Cucumis sativus* L. *Solanum lycopersicum* L.	Cucumber Tomato	Bačić et al., 2025 Bačić et al., 2023, 2025
	Slovenia	Asteraceae Solanaceae	*Senecio vulgaris* L. *Solanum lycopersicum* L.	Groundsel Tomato	Susič et al., 2020a Širca et al., 2004; Gerič Stare et al., 2018
South America	Brazil	Asteraceae	*Lactuca sativa* L.	Lettuce	Carneiro et al., 2014
			*Smallanthus sonchifolius* (Poepp.) H.Rob. *=Polymnia sonchifolia* Poepp.	Yacon	Carneiro et al., 2014
		Brassicaceae	*B. oleracea* L. *=B. oleracea* var. *italica* Plenck	Broccoli	Carneiro et al., 2014
		Cucurbitaceae	*Citrullus amarus* Schrad.	Watermelon	Carneiro et al., 2014
			*Cucumis sativus* L.	Cucumber	Carneiro et al., 2014
			*Luffa cylindrica* (L.) M.Roem.	Loofah sponge	Bellé et al., 2019
		Fabaceae	*Glycine max* (L.) Merr.	Soybean	Bellé et al., 2016
			*Phaseolus vulgaris* L.	Common bean	Carneiro et al., 2014; Machado et al., 2016
		Lamiaceae	*Lavandula angustifolia* subsp. *Angustifolia* *= L.spica* L.	Lavender	Carneiro et al., 2014
		Malvaceae	*Abelmoschus esculentus* (L.) Moench	Okra	Carneiro et al., 2014
			*Gossypium hirsutum* L.	Cotton	Carneiro et al., 2014
		Solanaceae	*Capsicum annuum* L.	Pepper	Carneiro et al., 2014
			*Nicotiana tabacum* L.	Tobacco	Carneiro et al., 2014
	Chile	Vitaceae	*Vitis vinifera* L.	Grapevine	Carneiro et al., 2014
	Guatemala	Solanaceae	*Solanum lycopersicum* L.	Tomato	Janssen et al., 2016; Gerič Stare et al., 2017

^1^ All scientific names were according to 'Plants of the World Online' (https://powo.science.kew.org/).

**Table 2:** Host status of cultivated crops to *Meloidogyne luci*.

Family	Scientific name	Common name	Host status^2^	References^2^
Amaranthaceae	*Beta vulgaris* L.	Beet	E	Şen and Aydınlı, 2021; Maleita et al., 2022a
	*B. vulgaris* subsp. *vulgaris*	Swiss chard		
	=*B. vulgaris* var. *cicla* L.		E	Strajnar et al., 2011
	=*B. vulgaris* var. *conditiva* Alef.		E,H	Strajnar et al., 2009
	*Spinacia oleracea* L.	Spinach	G,E,H	Strajnar et al., 2009, 2011; Şen and Aydınlı, 2021; Maleita et al.,2022a
Amaryllidaceae	*Allium cepa* L.	Onion	P,H	Strajnar et al., 2009; Şen and Aydınlı, 2021
	*A. porrum* L.	Leek	P	Şen and Aydınlı, 2021
Apiaceae	*Apium graveolens* L.	Celery	H	Strajnar et al., 2009
	*Daucus carota* L.	Carrot	P,H	Strajnar et al., 2009; Şen and Aydınlı, 2021; Maleita et al., 2022a
	*Foeniculum vulgare* Mill. =*F. vulgare* var. *azoricum* (Mill.) P.Fourn.	Florence fennel	H	Strajnar et al., 2009
	*Petroselinum crispum* (Mill.) Fuss	Plain-leaf parsley	P	Şen and Aydınlı, 2021
Asteraceae	*Cichorium endivia* L.	Endive	H	Strajnar et al., 2009
	*C. intybus* subsp. *Intybus* =*C. intybus* covar. *foliosum* (Hegi) Holub	Chicory	H	Strajnar et al., 2009
	*Helianthus annuus* L.	Sunflower	G,H	Strajnar et al.,2009; Şen and Aydınlı, 2021
	*Lactuca sativa* L.	Lettuce	P, G, E,H	Strajnar et al., 2009; Şen and Aydınlı, 2021; Maleita et al., 2022a
Brassicaceae	*Brassica cretica subsp. cretica* *=B. oleracea* var. *botrytis* L.	Cauliflower	P,H	Şen and Aydınlı, 2021; Strajnar et al., 2011, 2009
	*B. oleracea* L. *=B. oleracea* var. *acephala* DC.	Cabbage	P,G	Şen and Aydınlı,2021; Maleita et al., 2022a
	* =B. oleracea* var. *capitata* L.	Cabagge	H	Strajnar et al., 2009
	* =B. oleracea* var. *capitata* L. (alba)	White cabbage	P,G	Şen and Aydınlı, 2021
	* =B. oleracea* var. *capitata L.* (rubra)	Red cabbage	G	Şen and Aydınlı, 2021
	* =B. oleracea* var. *gemmifera* DC.	Brussels sprouts	G	Şen and Aydınlı, 2021
	* =B. oleracea* var. *gongylodes* L.	Kohlrabi	G,H	Strajnar et al., 2009, 2011
	* =B. oleracea* var. *italica* Plenck	Broccoli	P,H	Strajnar et al., 2009; Şen and Aydınlı, 2021
	* =B. oleracea* var. *sabauda* L.	Tronchuda cabbage	G,H	Strajnar et al., 2009, 2011
	*B. rapa* L.	Turnip	G	Şen and Aydınlı, 2021
	*Eruca sativa Mill.* *=E. vesicaria* subsp. *sativa* (Mill.) Thell.	Arugula	P	Şen and Aydınlı, 2021
	*Lepidium sativum* L.	Garden cress	G	Şen and Aydınli, 2021
	*Mustarda nigra* (L.) Bernh. =*Brassica nigra* (L.) W.D.J.Koch	Black mustard	G	Şen and Aydınlı, 2021
	*Raphanus raphanistrum* subsp. *sativus* (L.) Schmalh.	Radish		
	=*R. sativus* L.		P,E	Şen and Aydınli, 2021
	* =R. sativus* var. *radicula*		H	Strajnar et al., 2009
Convolvulaceae	*Ipomoea grandifolia* (Dammer) O'Donell	Sweet potato	G	Kaspary et al., 2021
	*I. hederifolia* L.		G	Kaspary et al., 2021
	*I. nil* (L.) Roth		G	Kaspary et al., 2021
	*I. purpurea* (L.) Roth		G	Kaspary et al., 2021
	*I. quamoclit* L.		G	Kaspary et al., 2021
Crassulaceae	*Hylotelephium spectabile* (Boreau) H.Ohba	Sedum	H	Carneiro et al., 2014
Cucurbitaceae	*Citrullus amarus* Schrad.	Watermelon	P	Carneiro et al., 2014; Fullana et al., 2024
	*Citrullus lanatus* (Thunb.) Matsum. & Nakai	Citron melon	P,G,E	Şen and Aydınlı, 2021; Maleita et al., 2022a; Fullana et al., 2024
	*Cucumis melo* L.	Melon	P,G,E,H	Strajnar et al., 2009; Şen and Aydınlı, 2021; Maleita et al., 2022a
	*Cucumis metuliferus* Jacques	Bitter wild cucumber	G	Fullana et al., 2024
	*Cucumis sativus* L.	Cucumber	E,H	Strajnar et al., 2009; Şen and Aydınlı, 2021; Fullana et al., 2024
	*Cucurbita maxima* Duchesne	Squash	G,E	Aydınlı et al., 2019
	*Cucurbita moschata* Duchesne	Crookneck squash	E	Aydınlı et al., 2019; Şen and Aydınlı, 2021; Maleita et al,. 2022a
	*Cucurbita pepo* L.	Pumpkin	E	Şen and Aydınlı, 2021; Maleita et al., 2022a
	*Luffa cylindrica* (L.) M.Roem.	Loofah sponge	E	Bellé et al., 2019
Fabaceae	*Cicer arietinum* L.	Chickpea	G,E,H	Şen and Aydınlı, 2021; Kefelegn et al., 2025
	*Glycine max* (L.) Merr.	Soybean	E	Bellé et al., 2016
	*Lathyrus oleraceus* Lam. *=Pisum sativum* L.	Pea	G,E,H	Strajnar et al., 2009; Şen and Aydınlı, 2021; Maleita et al., 2022a
	*Medicago sativa* L.	Alfalfa	H	EPPO, 2017
	*Phaseolus vulgaris* L.	Common bean	G,E,H	Strajnar et al., 2009; Bellé et al., 2019; Şen and Aydınlı, 2021
	*Vicia faba* L.	Faba bean	E	Şen and Aydınlı, 2021; Maleita et al., 2022a
Malvaceae	*Gossypium hirsutum* L.	Cotton	P	Carneiro et al., 2014
Passifloraceae	*Passiflora edulis* Sims	Passion fruit	P	Maleita et al., 2022a
Poaceae	*Hordeum vulgare* L.	Barley	G,H	Strajnar et al., 2009; Şen and Aydınlı,2021
	*Oryza sativa* L.	Rice	G	Şen and Aydınlı, 2021
	*Sorghum bicolor* x *S. sudanense*	Sorghum-sudangrass	P	Şen and Aydınlı, 2021
	*Triticum aestivum* L. =*T. vulgare*	Wheat	G	Şen and Aydınlı, 2021
	*x Triticosecale* Wittmack	Triticale	G	Şen and Aydınlı, 2021
	*Zea mays* L.	Maize	P,G,H	Strajnar et al., 2011; Şen and Aydınlı, 2021; Maleita et al., 2022a
	*Zea mays* subsp. *Mays* *=Z. mays* subsp. *saccharata* (Sturtev.) Zhuk.	Argentine pop corn	H	Strajnar et al., 2009
Polygonaceae	*Fagopyrum esculentum* Moench	Buckwheat	H	EPPO, 2017
	*Rumex patientia* L.	Curled dock	H	Strajnar et al., 2009
Solanaceae	*Capsicum annuum* L.	Pepper	P,G,E	Carneiro et al., 2014; Şen and Aydınlı, 2021; Maleita et al., 2022a
	*Nicotiana tabacum* L.	Tobacco	E	Şen and Aydınlı, 2021; Carneiro et al., 2014
	*Solanum lycopersicum* L.	Tomato	N,P,E,H	Strajnar et al., 2009, 2011; Aydınlı and Civelek, 2018; Kaspary et al., 2021; Şen and Aydınlı,2021; Maleita et al., 2022a; Bačić et al., 2023
	*S. melongena* L.	Eggplant	E,H	Strajnar et al., 2009; Sargin and Devran, 2021; Şen and Aydınlı, 2021
	*S. torvum* L.	Wild eggplant	N,P	Sargin and Devran, 2021
	*S. nigrum* L.	Black nighshade	H	Aydınlı and Mennan, 2016
	*S. tuberosum* L.	Potato	G,E	Maleita et al., 2018; Şen and Aydınlı, 2021

^1^ All scientific names were according to 'Plants of the World Online' (https://powo.science.kew.org/).

^2^ Host status categories based on reproduction factor (Rf = final population density / initial population density): N = Non-host (Rf = 0), P = Poor host (0 < Rf < 1), G = Good host (1 ≤ Rf < 10), E = Excellent host (Rf ≥ 10); H = No information available regarding Rf (Strajnar et al., 2009).

**Table 3:** *Meloidogyne luci* non-host plants.

Family	Scientific name	Common name	References
Amaryllidaceae	*Allium sativum* L.	Garlic	Şen and Aydınlı, 2021
Malvaceae	*Gossypium hirsutum* L.	Cotton	Şen and Aydınlı, 2021
Fabaceae	*Arachis hypogaea* L.	Peanut	Carneiro et al., 2014
	*Glycine max* (L.) Merr.	Soybean	Şen and Aydınlı, 2021
Poaceae	*Avena sativa* L.	Oat	Şen and Aydınlı, 2021
Rosaceae	*Fragaria x ananassa* (Duchesne ex Weston) Duchesne ex Rozier	Strawberry	Strajnar et al., 2009; Şen and Aydınlı,2021
Solanaceae	*Solanum torvum* Sw.	Wild eggplant	Sargin and Devran, 2021

First reports of this species, across the world parasitizing vegetables, flowers and fruits, were registered in South America (Brazil and Chile) and Asia (Iran) (Carneiro et al., 2014). In Brazil, *M. luci* was found attacking roots of lavender (*Lavandula angustifolia* subsp. *angustifolia*), cucumber (*Cucumis sativus* L.), lettuce (*Lactuca sativa* L.), broccoli (*Brassica oleracea* var. *italica* Plenck), okra [*Abelmoschus esculentus* (L.) Moench], common bean (*Phaseolus vulgaris* L.), yakon [*Smallanthus sonchifolius* (Poepp.) H. Rob.], kiwi fruit [*A. chinensis* var. *deliciosa* (A. Chev.) A. Chev.], soybean plants [*Glycine max* (L.) Merr.], and loofah sponge [*Luffa cylindrica* (L.) M. Roem.] (Carneiro et al., 2014; Machado et al., 2016; Bellé et al., 2016, 2019). In Chile, it was detected on grapevine (*Vitis vinifera* L.)*;* and in Iran, on rose (*Rosa* sp.), snapdragon (*Antirrhinum majus* L.) and sedum [*Hylotelephium spectabile* (Boreau) H. Ohba] (Carneiro et al., 2014; Janssen et al., 2016; Machado et al., 2016; Bellé et al., 2016, 2019). According to the mitochondrial coding genome analysis, this species was also reported in Guatemala (Janssen et al., 2016).

In Europe, first report of *M. luci* (previously identified as ‘*M. ethiopica’*) was in 2003 in Slovenia (Dornberk) on tomato (*Solanum lycopersicum* L.) (Širca et al., 2004). At that time, all infected plants were destroyed, and the nematode was not detected again for more than a decade. In Slovenia, *M. luci* was detected again in 2015, near Ljubljana (Širca et al., 2004; Strajnar et al., 2009; Gerič Stare et al., 2017, 2018). In 2009, *M. luci* was found on outdoor crop’s maize (*Zea mays* L.) and kiwifruit (*A. chinensis* var. *deliciosa*) in Greece (near Kavalla) and was reported on tomato in Italy (Pontecagnano), but details are lacking on its prevalence and distribution (Conceição et al., 2012; Maleita et al., 2012; Gerič Stare et al., 2017). In 2013, *M. luci* was found on potato (*Solanum tuberosum* L.) and in 2019 on tomato, the ornamental plant *Cordyline australis* (G. Forst.) Endl. and the weed *Oxalis corniculata* L. in mainland of Portugal (Maleita et al., 2018; Santos et al., 2019). In Azores Island of Portugal, *M. luci* was once again identified in association with potato (Rusinque et al., 2021). More recently, in 2021 and 2022, *M. luci* was identified in tomato and cucumber greenhouses of Vojvodina province, Serbia (Bačić et al., 2023, 2025). In 2024, *M. luci* was detected at a new location in Slovenia, this time on tomatoes cultivated in open field conditions (Susič et al., 2026a). This finding represents the first report of *M. luci* in open field production systems in Slovenia, as previous detections were limited to greenhouse-grown tomatoes.

Outside of Europe, this species was detected in Asian part of Türkiye on tomato greenhouses of the University of Ondokuz and in cucumber greenhouses in Çarşamba district, Samsun province (Aydınlı and Mennan, 2016; Aydınlı et al., 2013; Gerič Stare et al., 2017). Lately, there are new records of this nematode in vegetable fields and kiwi orchards of Samsun Province (Aydınlı, 2018; Aydınlı and Mennan, 2022). The latest record of *M. luci* was in Africa (Ethiopia) on chickpea (*Cicer arietinum* L.) (Kefelegn et al., 2025).

*Meloidogyne luci* may have been misidentified as *M. ethiopica* in several surveys, as was confirmed for European and Turkish populations (Gerič Stare et al., 2017). Furthermore, it has been demonstrated that the primer pair MI-F/MI-R, commonly used for the identification of *M. incognita*, considered the most globally widespread and damaging plant-parasitic nematode in the world can also amplify DNA from *M. luci*. This means that cross-reaction may occur and false-positive identification of *M. incognita* may be obtained when *M. luci* is actually present in the sample (Bačić et al., 2025). These findings further suggest that the specificity of the MI-F/MI-R primer pair is not absolute and that caution should therefore be exercised when interpreting results obtained using this diagnostic marker. Consequently, some records previously attributed to *M. incognita* in monitoring programs conducted in past decades may in fact correspond to *M. luci.*

**Figure 1:**
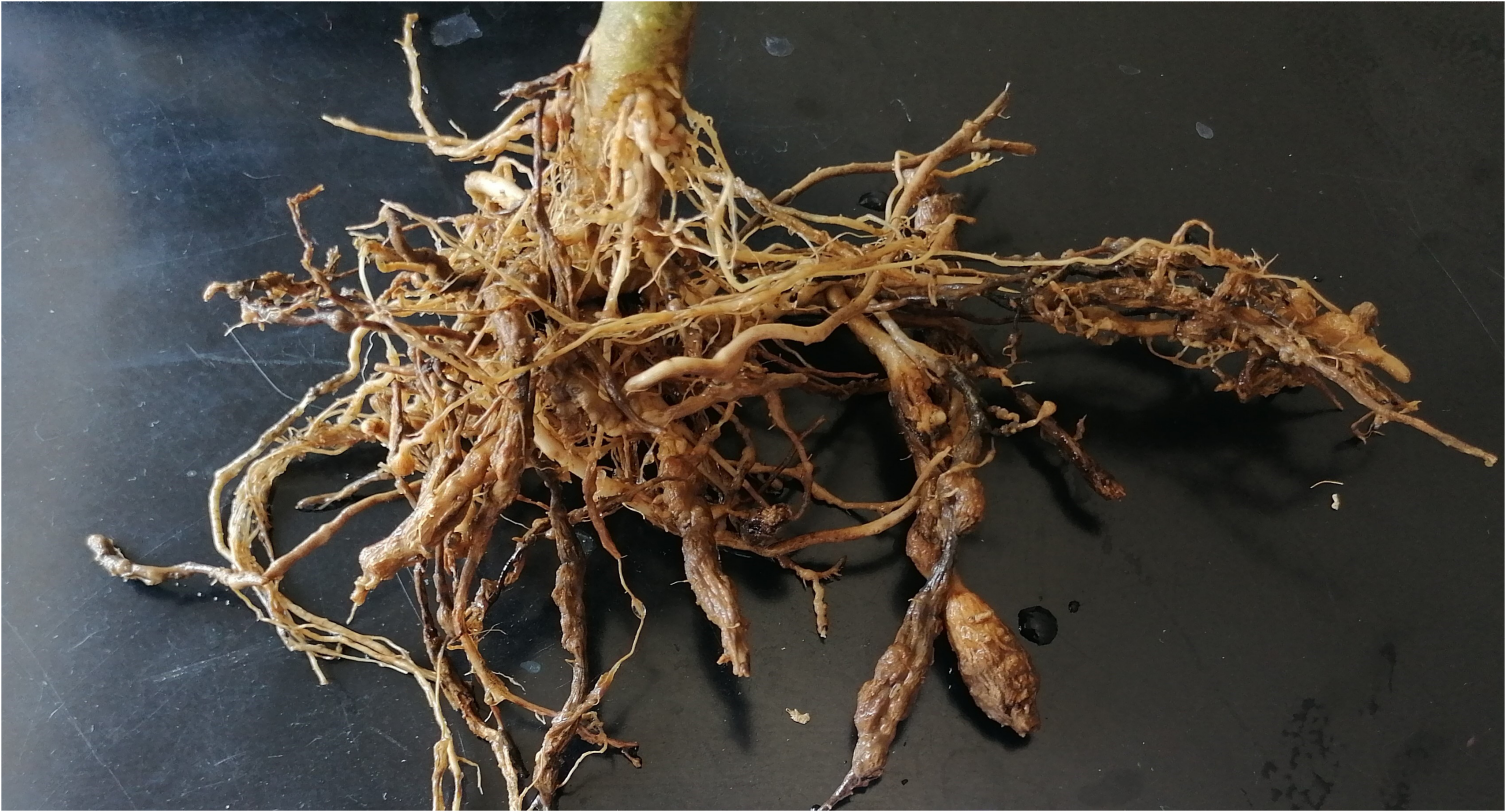
Extensive root galls observed on tomato (*Solanum lycopersicum* L.) roots caused by the RKN species *Meloidogyne luci* during an official survey for quarantine RKN in Serbia (village of Lugovo - 430 43’32,562; 190 08’55,168, Province Vojvodina), in August 2021 (Bačić et al., 2023). Reprinted from Bačić et al., 2023, First Report of the Root-Knot Nematode *Meloidogyne luci* on Tomato in Serbia, Plant Disease 107:2554.

Plants infected with *M. luci* show nonspecific symptoms, similar to those exhibited by plants infected by other RKN species, which include yellowing, wilting, and stunted growth of above-ground, while below ground symptoms can be recognized by extensive root galling, affecting the development of root system (**Figure 1**) (Širca et al., 2004; Aydınlı et al., 2013; Machado et al., 2016; Bellé et al., 2016; Strajnar et al., 2009; Santos et al., 2019; Bačić et al., 2023). Sometimes, symptoms may not be observable in the plants, probably due to low population density. In Portugal, potato plants infected with *M. luci* did not show symptoms of RKN infection (Rusinque et al., 2021). Further, Žibrat et al. (2021) showed that *M. luci* may develop a latent infestation without visible symptoms on the potato tubers. Such latent infestation may pose a high risk for uncontrolled spread of the pest, especially via seed potato. On the other hand, considerable damages caused by *M. luci* were reported in Portugal, Slovenia and Türkiye (Gerič Stare et al., 2018; Maleita et al., 2018; Aydınlı et al., 2019; Şen and Aydınlı, 2021; Maleita et al., 2022a; Aydınlı and Mennan, 2022). In Slovenia, more than 80% of tomato plants were severely damaged (Gerič Stare et al., 2018) and in Türkiye, *M. luci* was the most prevalent RKN species, reported in 74% of the kiwi orchards sampled (Aydınlı and Mennan, 2022).

## 3 Identification

### 3.1 Morphological analysis

*Meloidogyne* species can be differentiated by the combination of the perineal pattern and stylet morphology of females, stylet, head and tail morphology of males and head and tail morphology of second-stage juveniles (J2) (Hunt and Handoo, 2009). But identification based on morphological characters of adults and J2 is unreliable, it implies a laborious microscopic examination, which relies on measurements and comparison of structures, and requires enormous experience and expertise due to inter- and intra-specific variability and the frequent occurrence of more than one species in the same sample (Blok and Powers, 2009). These problems led researchers to use biochemical and molecular methods to confirm and complement nematode species identification.

**Figure 2:**
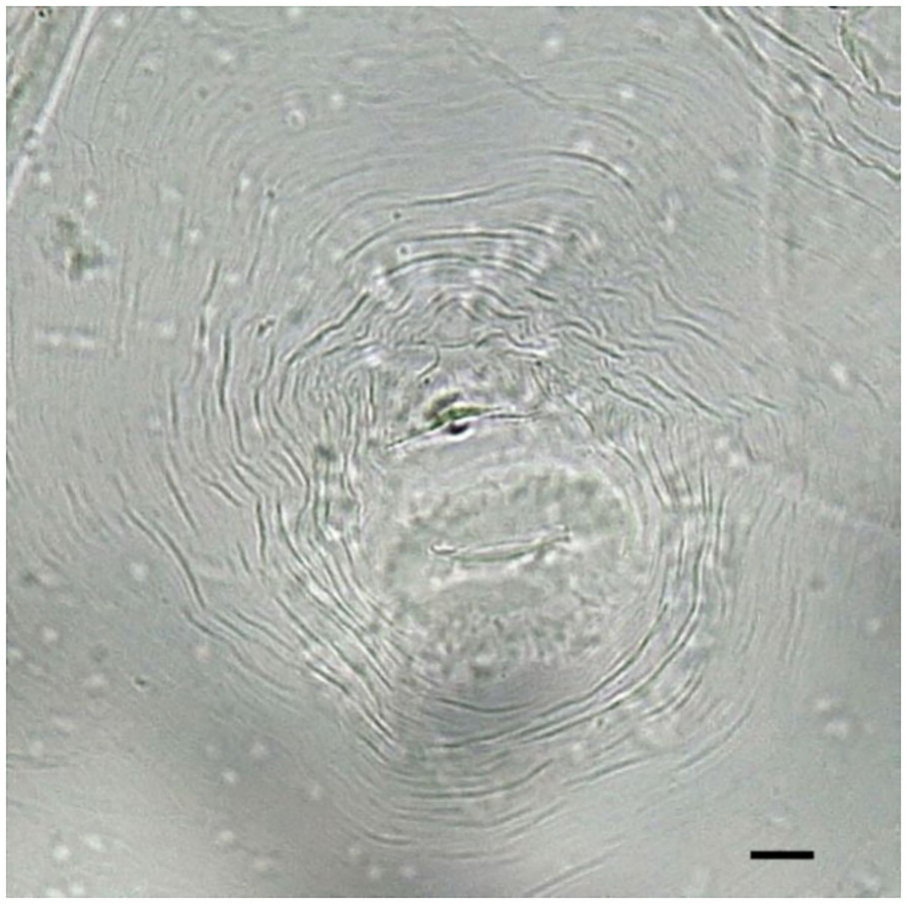
Perineal patterns of females of a *Meloidogyne luci* isolate from Serbia, characterized by oval to squarish shapes, smooth and wavy striae, weakly defined lateral lines, and a rounded dorsal arch. Scale bar 10 µm (Bačić et al., 2023). Reprinted from Bačić et al., 2023, First Report of the Root-Knot Nematode *Meloidogyne luci* on Tomato in Serbia, Plant Disease 107:2554.

According to Carneiro et al. (2014); Bellé et al. (2016); Maleita et al. (2018) and Rusinque et al. (2021), *M. luci* females are oval or pear-shaped with prominent neck; body cuticle annulated; head region barely separated from the body; stylet strong with knobs well distinctive. Shape of female patterns is highly variable, oval to squarish like *M. ethiopica* with a low to moderately high dorsal arc without shoulders (**Figure 2**). Dorsal striae wavy to smooth, generally distinctly, without the presence of striae in perivulval region; striae sometimes present on lateral parts of vulva and tail terminus occasionally apparent. Distinctive lateral lines are absent or weakly defined. Phasmids are small, right opposite and posterior to anus. Males are vermiform, body cuticle annulated; head region not set apart from the body; stylet robust with knobs rounded; tail short with curved spicules; lateral field with four incisures. The J2 are vermiform and slender. The head region slightly set apart from the body; stylet with small oval knobs; tail conoid with distinctive hyaline terminus; excretory pore well defined; phasmids small, found posterior to anus.

### 3.2 Biochemical analysis

Biochemical electrophoretic analysis of non-specific esterases (EST) remains the first step in the RKN species identification process, when young egg-laying females are available. This sensitive and efficient method is very useful in the detection of populations with more than one species that can be easily separated to obtain pure isolates, by being able to use a protein extract from only one female. The extract proteins are separated by electrophoresis in separating (pH 8.8) and stacking (pH 6.8) vertical polyacrylamide gels, at 7% and 3%, respectively (Maleita et al., 2012). The gels are stained for EST activity with the substrate α-naphthyl acetate (Maleita et al., 2012). Protein extract of *M. javanica* young egg-laying females is always included in each gel as a reference for comparison and RKN species identification. Phenotypes are designated with a letter(s) suggesting the nematode species, followed by a number indicating the number of bands (Esbenshade and Triantaphyllou, 1985).

In the *M. luci* isolates, three bands of EST activity are detected, corresponding to the phenotype L3, distinct from other *Meloidogyne* species (Carneiro et al., 2014; Gerič Stare et al., 2017; Maleita et al., 2018; Rusinque et al., 2023). Nevertheless, the EST phenotype of *M. luci* resembles EST phenotype of *M. ethiopica* (E3) and for this reason all European and Turkish *M. ethiop*ica populations reported before 2017 were reclassified as *M. luci* (Gerič Stare et al., 2017). Esterase phenotype of both species usually shows three bands and only differ by the position of first band in relation to *M. javanica* reference (J3). Note that *M. ethiopica* population with two bands of the EST profile (E2) has also been documented (Gerič Stare et al., 2017). The first band of *M. luci* is located at the same level of the first band of *M. javanica*, whereas for *M. ethiopica* the first band is located above (Carneiro et al., 2014; Gerič Stare et al., 2017; Maleita et al., 2018; Rusinque et al., 2023). Considering the great similarity between L3 and E3 phenotypes, it is advisable to include in each gel a *M. luci* or/and *M. ethiopica* reference isolate for comparison and for a more reliable identification.

The time-consuming nature of this methodology, together with its requirement for fully developed females, limits its applicability, particularly when large numbers of samples received in laboratories consist of soil or deteriorated root material. In addition, the high similarity of esterase phenotypes among closely related species, especially between *M. luci* and *M. ethiopica*, may complicate reliable identification when biochemical data are used alone. Unlike morphological approach, which requires multiple developmental stages, and biochemical approach which requires mature females for reliable identification, molecular methods can be applied to different types of nematode material, including eggs, J2, females and males. This makes molecular tools particularly useful when only eggs or juvenile stages are available in the sample. Therefore, molecular tools became necessary to improve the accuracy, sensitivity and reliability of species identification, particularly for the differentiation of closely related *Meloidogyne* species and for the analysis of samples containing low amounts of nematode material or mixed populations.

### 3.3 Molecular analysis

In addition to morphological and biochemical similarities*, M. luci* shares molecular similarities with *M. ethiopica*, to such an extent that European *M. luci* isolates were misidentified as *M. ethiopica*, prior to the *M. luci* description (Gerič Stare et al., 2017). Their molecular relationship has been investigated using different DNA regions: (ITS1) rRNA and D2-D3 fragment of 28S rRNA regions (Carneiro et al., 2014; Machado et al., 2016); mitochondrial DNA (mtDNA) cytochrome oxidase subunit II (COII) (Gerič Stare et al., 2017; Maleita et al., 2018) and ITS1-5.8S-ITS2 rRNA region and cytochrome oxidase subunit I (COI) mtDNA region (Maleita et al., 2018). The ITS and D2-D3 fragments of the 28S rRNA region were shown to be unsuitable for examining the connection among these closely related RKN species (Gerič Stare et al., 2017; Maleita et al., 2018). The usefulness of mtDNA markers for tropical RKN differentiation was reported by Gerič Stare et al. (2017); Janssen et al. (2016); Maleita et al. (2018) and Kefelengn et al. (2026). According to Gerič Stare et al. (2017) the phylogenetic analysis of the COII/16S rRNA region of mtDNA showed that *M. luci* formed a monophyletic clade and allowed a clear separation of this species from other RKN. Consequently, several tests have been developed and validated for *M. luci* and *M. ethiopica*. Species-specific SCAR-PCR tests are available for *M. ethiopica* (Correa et al., 2014) and *M. luci* (Maleita et al., 2021) and a real-time PCR test (Žibrat et al., 2021) has been designed for the identification of species within the *M. ethiopica* group (MEG: *M. ethiopica*, *M. luci* and *M. inornata*). A specific amplification product on *M. luci* was acquired from a random amplified polymorphic DNA (RAPD) analysis (Maleita et al., 2021). It was based on the intraspecific variability found in RAPD markers and converted into a sequence characterized amplified region (SCAR) marker. After the *M. luci* specific DNA fragment was sequenced, species-specific primers Mlf (5’- ACT CCT GCG ACC TCA TGG CAT TTA -3’) and Mlr (5’-ACT CCT GCG AAC ACA ACA TTT ACT-3’) were designated, and applied on molecular detection of *M. luci* on the galls of infected roots (Maleita et al., 2021, **Supplementary Table 1**). The same methodology had already been developed to design the *M. ethiopica*-specific primers meth-F (5′-ATG CAG CCG CAG GGA ACG TAG TTG-3′) and meth-R (5′-TGT TGT TTC ATG TGC TTC GGC ATC-3′) (Correa et al., 2014). However, problems with specificity of this later test for *M. ethiopica* have been observed (Aydınlı and Mennan, 2016; dos Santos et al., 2019; Barbara Gerič Stare, *personal communication*).

Morphometrical and molecular characters, namely the structure of *map*-1 genes, of *M. ethiopica*, *M. luci* and *M. inornata* isolates were similar and distinct from other tropical RKN species, supporting the close relationship of these species and therefore, they were classified together as *M. ethiopica* group (Gerič Stare et al., 2019). Consequently, a PCR based method using specific primers to the *M. ethiopica* group was developed, targeting the mtDNA region between the COII and 16S rRNA genes: primers Me309F (5’-CTA ATT TGG GTG AAT TT-3’) and Me549R (5’-AAT CAA AAT CTT CTC CT-3’) (Gerič Stare et al., 2019; **Supplementary Table 2**). In the same research work, specific primers for the tropical RKN group species were also designed: reverse primer Mt575R (5’-AGA ACT TAA ACT CTA AAT AAC-3’) in combination with forward primer C2F3 (5’-GGT CAA TGT TCA GAA ATT TGT GG-3’), described previously by Powers and Harris (1993) (Gerič Stare et al., 2019; **Supplementary Table 3**). Nevertheless, in a consensus phylogenetic tree inferred from COI gene and *COII*-16S rRNA sequence grouped in the same clade RKN species belonging to the *M. ethiopica* group and the RKN tropical species (Álvarez-Ortega et al., 2019). Similarly, phylogenetic reconstruction based on the sequence alignments of common benchmarking universal single copy orthologs (BUSCO) from selected species/populations of RKN genomes publicly available in the GenBank collection showed the MEG species formed a separate clade within tropical RKN species. Though closely related, *M. luci* separated from *M. ethiopica* and *M. inornata* (Susič, 2020). Susič (2020) showed that sequence comparison and phylogenetic analyses of the D2-D3 region of the 28S rDNA, ITS rDNA, and mtDNA (*Nad5*, *cox2* and *cox1*) revealed limited resolution in distinguishing *M. luci* from closely related species, such as *M. ethiopica, M. inornata* and *M. hispanica*, underscoring the challenges of differentiating these taxa using partial gene sequences. Only the full mitogenome sequencing provided clear separation of *M. luci* from *M. hispanica* and its closest relatives, confirming its diagnostic power (Susič, 2020). In 2021, using hyperspectral imaging and a molecular approach to detect RKN DNA with real-time PCR, based on Me309F and Me549R primers, it was possible to detect *M. luci* in symptomatic as well as in asymptomatic infected potato tubers (Žibrat et al., 2021). However, this approach is not specific to *M. luci* and the test would need to be combined or substituted with a *M. luci* specific test (e.g. Maleita et al., 2021) to prevent uncontrolled spread of *M. luci* via infected potato tubers.

Additional tools, such as high-throughput Kompetitive Allele-specific PCR (KASP) assay was already developed for the identification of *M. luci* (Devran and Goknur, 2025). With this assay *M. luci* was easily separated from *M. inornata* and other RKN species (*M. arenaria*, *M. chitwoodi*, *M. hapla*, *M. incognita*, and *M. javanica*). However, KASP assay was not validated with *M. ethiopica*, which may give more information about the specificity of the assay (Devran and Goknur, 2025).

Long-read Pacific Biosciences Sequel and short-read Illumina HiSeqX sequencing data were already used to produce a high-quality *M. luci* genome assembly (accession ERS3574357; DDBJ/ENA/GenBank). This assembly of *M. luci* population SI-Smartno V13 can be used to determine the correct phylogenetic position of the clade, identification of genetic changes related to the origins of virulence, and in the study of evolutionary history of this organism (Susič et al., 2020b).

To conclude, DNA suitable for molecular tests can be extracted from single juveniles, females and males, or from small root galls or egg masses, using standard nematode lysis protocols. Then, a two-step PCR approach can be used for the molecular identification of *M. luci*. First, amplification is performed using the *M. ethiopica* group-specific primers Me309F and Me549R; samples yielding a 241 bp product are subsequently subjected to a second PCR with the *M. luci*-specific primers Mlf/Mlr. The presence of *M. luci* is confirmed by the amplification of an approximately 770 bp fragment (Gerič Stare et al., 2019; Maleita et al., 2021) (**Supplementary Tables 1**-**S3**). This methodology can be adopted in routine inspections and is very useful for monitoring distribution and spread of this emerging plant pathogen. The advantage of the two-step PCR approach, compared with a single *M. luci*-specific PCR, is that it also provides information on the potential presence of the closely related quarantine species *M. ethiopica* and exhibits higher sensitivity, as the initial group-specific amplification step enables the detection of lower levels of target DNA that might not be detected by species-specific primers alone (Carla Maleita and Barbara Gerič Stare, *personal communication*).

### 3.4 Differential hosts

Historically, identification of RKN relied not only on morphological observations but also on biological assays, particularly differential host tests, which were used to distinguish species based on their ability to reproduce on selected host plants (Sasser et al., 1983). However, due to limitations in reliability, time consumption and overlap in host responses among closely related species, such approaches have largely been replaced or complemented by biochemical and molecular diagnostic methods.

*M. luci* and *M. ethiopica* are closely related species at the molecular level and exhibit a high degree of similarity in morphological and biochemical characters, which has historically led to misidentifications. In addition to their molecular and phenotypic resemblance, both species share overlapping host range and occupation of similar ecological niches, parasitizing many of the same cultivated and wild plant species. To date, no differential host plant has been identified that would reliably distinguish between *M. luci* and *M. ethiopica* (Gerič Stare et al., 2017).

## 4 Bio-ecology

Symptoms exhibited by RKN infected plants caused by an altered plant metabolism, usually involving weakness of the root systems and leaf nutritional deficiencies, compromise plant development and lead to decreased production. The effects of increasing inoculum levels of *M. luci* on nematode reproduction and growth of tomato, in pot experiments, was studied by Aydınlı and Civelek (2018). The increase in inoculum level caused reduction of plant growth parameters, except for root weight, and a significant increase in the number of galls (Aydınlı and Civelek, 2018). Already in 2012, studies of Strajnar et al. (2012) on effect of *M. luci* (previously identified as ‘*M. ethiopica’*) parasitism on water management and physiological stress in tomato showed that root gall formation had negative impact on water transportation in infected plants causing water stress. Additionally, it led to physiological stress associated with decreased stomatal conductivity, transpiration, and photosynthesis (Strajnar et al., 2012).

Despite their probable tropical origin, according to the assessment of the potential suitability for *M. luci* survival and development, by using CLIMEX during the MeloTrop Euphresco Project, *M. luci* could survive outdoor in the most Mediterranean countries (Širca et al., 2021). The base temperature (Tb) and thermal constant (S) of *M. luci* were determined and the relative lower Tb of 6.11 °C, combined with the high number of S (555.6) required to develop and successfully reproduce, confirmed that this species can adapt/is adapted to cold climatic conditions (Duarte Santos, *personal communication*). These findings are in accordance with that of Hassen Eslah (2023). At 12 °C, regardless of the incubation period (2 to 10 weeks) in the absence of a host, more than 70% of *M. luci* J2 survived, very similar to those obtained at 4 °C, except for 10 weeks of incubation (18% survival). However, infectivity and reproduction decrease significantly when compared to the temperate RKN *M. chitwoodi* (Hassen Eslah, 2023).

Therefore, the spread of *M. luci* across Europe does not appear to be limited by temperature, indicating a potential to extend into a wider range of regions than previously anticipated. *Meloidogyne luci* was detected in open fields of corn, cucumber, kiwi, potato, tomato of Greece, Portugal, Slovenia and Türkiye, and there are also reports of *M. luci* survival in open fields in sub-Mediterranean and continental regions of Slovenia, despite soil temperatures below zero during winter (Strajnar et al., 2011; Conceição et al., 2012; Ayndili et al., 2013; Aydınlı and Civelek, 2018; Maleita et al., 2018; Susič et al., 2026a). Hence, *M. luci* represents a significant threat to both open-field and greenhouse cultivation systems across Europe and globally. The ability of this species to spread to new geographic regions is limited by their low mobility in the soil. Thus, the most likely mode of spread of these pests to new areas is through infected soil or plant material from regions where this nematode is present. Likewise, higher temperatures due to climate change might cause an increase in the number of generations of nematodes, leading to an increased level of economic damage.

## 5 Integrated management strategies

Once RKN are established in the soil their control or eradication is very difficult. Currently, to promote sustainable food production and protect human health and the environment, the main objective of RKN management strategies is to increase crop yield by reducing the nematode population in soil, limiting the damage to a level economically acceptable, with minimum impact on the environment. The most common RKN management strategies include cultural practices (e.g. crop rotation, fallow, trap crops, solarization, sanitation), genetic resistance, biological control, and organic amendments and chemical treatment.

Historically, the control of RKN has predominantly relied on the use of synthetic nematicides. However, many nematicides, based on e.g. ethylene dibromide (EDB), dibromochloropropane, and methyl bromide, are being removed from the market due to the adverse effects on the environment and human health and therefore, the availability of effective products for RKN management is becoming limited (Chitwood, 2002). Consequently, the search for effective, sustainable, and environmentally friendly alternatives to synthetic nematicides, such as plant-based solutions, was stimulated (Zasada et al., 2010). Simultaneously, the use of cultural methods, host resistance and biological control are gaining importance in the integrated management of RKN, despite their limitations.

The successful eradication of *M. luci* in Slovenia at location Šmartno highlights that, despite the difficulty of control, a strategic combination of chemical, cultural, and host-resistance approaches can lead to complete elimination of RKN under well-managed conditions (Širca et al., 2024).

### 5.1 Crop rotation

The use of crop rotation requires knowledge about the host status of a large number of plants. This approach is relatively straightforward for species with a narrow host range, but it becomes more limited for those with a broad host range.

The host status of several cultivated plants to *M. luci* was evaluated and substantial differences occurred among plant species and cultivars (Strajnar et al., 2009; Şen and Aydınlı, 2021). Şen and Aydınlı (2021) demonstrated that from the 71 cultivars evaluated, 46 were classified as good (1 ≤ Rf < 10) or excellent hosts (Rf ≥ 10). Only five cultivars were identified as non-hosts (Rf = 0), and suitable for crop rotation schemes: strawberry cv. Sweet Ann, cotton cv. SC 2079, garlic cv. Taşköprü, soybean cv. Arısoy and oat cv. Even. However, if cotton and soybean were classified as non-host by Şen and Aydınlı, 2021, they were considered as poor host and excellent host, respectively, by Carneiro et al. (2014) and Bellé et al. (2016).

For citron melon, lettuce, melon and pepper, the Rf values varied, depending on the cultivar, from 0 < Rf > 10 and were classified as poor, good or excellent hosts (**Table 2**). Two potential rootstocks, *Cucumis metuliferus* BGV11135 and *Citrullus amarus* BGV5167, were also tested in pot experiments with *M. luci* (Fullana et al., 2024). Lower numbers of galls (G), egg masses (EM), and eggs per plant were reported than their respective susceptible plants and could be classified as poor hosts. On the other hand, three cultivars of *Cucumis sativus* tested were classified as excellent hosts for *M. luci* (Şen and Aydınlı, 2021; Fullana et al., 2024). *Citrullus lanatus* was classified as excellent host for *M. luci* by Fullana et al., 2024. Cultivars Charleston Gray and Sugar Baby were good hosts for *M. luci* (Rf = 2.52-2.7); whereas cv. Crimson Sweet was a poor host (Rf = 0.51) (Şen and Aydınlı, 2021; Maleita et al., 2022a).

Additionally, cv. Bt Burbeyaz of radish was classified as poor host, while cv. Bt Burkir was an excellent host (Şen and Aydınlı, 2021). An unidentified cultivar of maize and cvs. Otello and Sy Lucroso were reported as good hosts, whereas cvs. Apex and Merit were found to be poor hosts for *M. luci* (Şen and Aydınlı, 2021; Maleita et al., 2022a).

Cultivated eggplant cvs. Faselis F1, Aydın Siyahı and Topan 374 were excellent hosts for *M. luci* (Sargin and Devran, 2021; Şen and Aydınlı, 2021). *Solanum torvum* rootstock cv. Hawk was classified as resistant for all *Mi-1.2*-virulent and avirulent isolates of *M. incognita*, *M. javanica*, and *M. luci*, according to the 0–5 egg mass index (EMI), with 0 < EMI < 1. Cultivar Boğaç was also found resistant to all species and isolates, except for the avirulent isolate of *M. luci* (TK4) considered as a non-host (EMI = 0) (Sargin and Devran, 2021). Cross-incompatibility between the cultivated *S. melongena* and the wild species *S. torvum* does not allow the development of resistant hybrids. So, grafting with resistant rootstocks appears as a valuable tool for controlling *M. luci* populations irrespective of their (a)virulence status to the *Mi-1.2* gene (Sargin and Devran, 2021).

The evaluation of the reproduction of *M. luci* and *M. ethiopica* on 20 bean cultivars showed that all tested cultivars were good to excellent hosts for *M. luci*, with Rf ranging from 6.0 to 39.94 (Bellé et al., 2019; Şen and Aydınlı, 2021). Similar results were obtained for chickpea and pea by Şen and Aydınlı (2021); Maleita et al. (2022a) and Kefelegn et al. (2025).

According to the studies on screening of *Cucurbita maxima* and *C. moschata* genotypes for resistance against *M*. *luci*, only genotypes 57SI21-O11, 55CA15-O9, 55CA15-A3 and G14-IP1 of *C. maxima* were considered good hosts (1 ≤ Rf < 10); the remaining genotypes were classified as excellent hosts (Rf > 10) (Aydınlı et al., 2019). The pathogenicity of *M. luci* on 16 commercial potato cultivars was studied by Maleita et al. (2018) and all were reported as excellent hosts. Nevertheless, Şen and Aydınlı (2021) classified cv. Agria of potato as a good host.

Briefly, several plant species/cultivars were recorded as excellent hosts (Rf ≥ 10) for *M. luci*, which include beet, chickpea, citron melon, common bean, crookneck squash, cucumber, eggplant, faba bean, lettuce, loofah sponge, melon, pea, pepper, potato, pumpkin, radish, soybean, spinach, squash, swiss chard, tobacco, and tomato (**Table 2**). Arugula, broccoli, cvs. Bacalan, Coração and Lombarda of cabbage, carrot, cv. Igloo of cauliflower, cv. Crimson Sweet of citron melon, cotton, leek, cvs. Bt Ivanka, Cocktail and Iceberg of lettuce, cvs. Apex and Merit of maize, cvs. Ananas, Bt Akhisar Topan 016 and Kırkağaç 637 of melon, onion, passion fruit, cvs. Bt Dik Dolma 016 of pepper, plain-leaf parsley, cv. Bt Burbeyaz of radish, sorghum-sudangrass, watermelon, cv. Bafra of white cabbage, and wild eggplant were classified as poor hosts (0 < Rf < 1) (**Table 2**).

As far as we know, only four species were considered non-hosts (EM = 0 and Rf = 0): *Allium sativum* L. (garlic), *Arachis hypogaea* L. (peanut), *Avena sativa* L. (oat) and *Fragaria x ananassa* (Duchesne ex Weston) Duchesne ex Rozier (strawberry) (**Table 3**). For cotton (*Gossypium hirsutum*), soybean (*Glycine max*), and wild eggplant (*Solanum torvum*) contradictory data was found (**Tables 2**, **3**).

In soils infested with *M. luci*, due to their wide host range, when devising an integrated nematode management by crop rotation it is crucial to take into account the knowledge about the host status of potential selected plant species and/or cultivars. So, nematode management by crop rotation should be performed locally and depend on the nematode species found in the field.

Within crop rotation systems, effective management of *M. luci* requires not only careful selection of cultivated crops based on their host status, but also consideration of non-crop vegetation present in the field. Weeds can function as alternative hosts and reservoirs, enabling nematode survival between susceptible crops and thereby reducing the effectiveness of rotation schemes. In particular, five species of the *Ipomoea* genus and *Senecio vulgaris* L. have been shown to support *M. luci* reproduction, highlighting their potential role in maintaining and amplifying populations (Kaspary et al., 2021; Susič et al., 2020a). Therefore, integrated crop rotation strategies should incorporate weed management to minimize the persistence and spread of *M. luci*.

### 5.2 Host plant resistance

The expression of plant resistance is characterized by suppression of nematode development and reproduction. In some host plants, multiple resistance genes have been identified but only some are available in cultivated crops (Abd-Elgawad, 2022). Tomato is one of the crops in which genetic resistance against RKN has been investigated and the *Mi-1* gene found to play an important role. In the 1940s, the RKN resistance gene was introgressed into the cultivated tomato *S. lycopersicum* from the wild species *S. peruvianum* accession PI-128657 using embryo rescue (Smith, 1944). Currently, many tomato cultivars available commercially carry the *Mi-1* gene.

This gene confers resistance to the three most common RKN species, *M. arenaria*, *M. incognita* and *M. javanica*, preventing the establishment of a functional feeding site able to support the nematode development and reproduction (Verdejo-Lucas et al., 2009). However, newly emerged natural isolates of these RKN species and other *Meloidogyne* spp. can overcome this resistance gene, and also *Mi*-mediated resistance breaks down at soil temperatures above 28°C for some *Meloidogyne* spp (Dropkin, 1969; Ammati et al., 1986; Tzortzakakis et al., 2014).

Several tomato cultivars were tested against *M. luci* and were classified from resistant to excellent hosts (Arslantaşlı et al., 2025; Aydınlı and Mennan, 2019; Gabriel et al., 2020; Santos et al., 2020; Maleita et al., 2022a). Tomato cv. Venezia with *Mi-1.2* resistance gene was reported as resistant to a *M. luci* isolate from Slovenia, before species description and *M. ethiopica* isolates reclassification (Strajnar and Širca, 2011; Gerič Stare et al., 2017). The ability of *M. luci* and *M. ethiopica* to reproduce on 27 tomato hybrids and cultivars was also assessed by Santos et al. (2020). From these, the *Mi-1.2* gene was identified in 15 tomato genotypes (two homozygous – MiMi - and thirteen heterozygous – Mimi - at the *Mi* locus), from which seven were reported as poor hosts (0.01 ≤ Rf ≤ 0.66), and one displayed a resistant reaction (‘Reconquista’) to both RKN species (Rf = 0). Tomato genotypes Valoasis RZ F1 and SV1917 were identified as resistant to *M. ethiopica* and *M. luci*, respectively (Santos et al., 2020). Gabriel et al. (2020) also demonstrated that tomato cv. Debora Plus (Mimi) is a poor host for *M. luci* and other 12 RKN species, including *M*. *arenaria*, *M*. *ethiopica*, *M*. *exigua*, *M*. *hispanica*, *M*. *incognita*, *M*. *inornata*, *M*. *izalcoensis M*. *javanica*, *M*. *konaensis*, *M*. *morocciensis*, *M*. *paranaensis*, and *M*. *petunia*, and an excellent host for *M*. *enterolobii* and *M*. *hapla.* These studies confirmed that *Mi-1.2* gene is effective to suppress *M. luci* reproduction, and the similar RKN species *M. ethiopica*, and can be used in integrated nematode management programs.

Nonetheless, the successful employment of tomato plants with the *Mi-1.2* gene in integrated control strategies could be compromised by the occurrence of nematode populations able to overcome this resistance gene (Aydınlı and Mennan, 2019; Arslantaşlı et al., 2025; Susič et al., 2026b). Four out of 37 *M. luci* RKN isolates from the middle Black Sea Region of Türkiye overcame the resistance conferred by *Mi-1.2* gene (Aydınlı and Mennan, 2019). This is the first report for resistance-breaking isolates of *M. luci* in tomato (Aydınlı and Mennan, 2019). Severe gall formation (4.3 ≤ Gall Index ≤ 5.0) was observed on 14 tomato cultivars and rootstocks inoculated with the *Mi*-virulent isolate Or-2 of *M. luci*, identified previously, and Rf values varied from 2.32 (Browny) to 48.64 (King Kong RZ) (Aydınlı and Mennan, 2019; Arslantaşlı et al., 2025).

Similarly, a virulent *M. luci* population was identified in greenhouse vegetable production in Slovenia, capable of reproducing on tomato cultivars carrying the *Mi-1.2* resistance gene. In a controlled pot experiment, comparable reproduction was observed on both resistant and susceptible tomato cultivars, confirming its resistance-breaking ability (Susič et al., 2026b).

Significant differences in *M. luci* reproduction were also detected between the *Mi* allelic conditions and tomato genotypes within *Mi* allelic conditions (Santos et al., 2020; Arslantaşlı et al., 2025). The *Mi-1.2* gene has greater impact in homozygous (MiMi) host genotypes at the *Mi* locus than on heterozygous (Mimi) genotypes (Santos et al., 2020; Arslantaşlı et al., 2025). In addition to the dosage effect of the *Mi-1.2* gene, an influence of the plant’s genetic background on *M. luci* reproduction was also demonstrated, as already reported for several tomato cultivars and RKN species (Maleita et al., 2011; Santos et al., 2020; Arslantaşlı et al., 2025). For example, all rootstocks and cultivars with *Mi-1.2* gene (MiMi) were poor hosts for a *M. luci* avirulent isolate (Rf < 1), except the King Kong RZ rootstock which was identified as a good host (Rf = 1.27).

Tomato plants with the *Mi-1.2* gene can be an option for *M. luci* control but should be included in an integrated pest management program, such as crop rotation, due to the occurrence of *M. luci* populations able to overcome the *Mi* resistance gene and as a repeated exposure to *Mi* tomato cultivars could lead to a selection of virulent isolates.

### 5.3 Chemical control

Chemical control options may contribute to the suppression of *M. luci* populations in protected cultivation systems. Certain soil-applied insecticidal or nematicidal products have been reported to reduce nematode multiplication under greenhouse conditions. The effects of some synthetic chemicals on *M. luci* reproduction have been already studied in Slovenia. No *M. luci* reproduction was detected in a pot experiment after soil treatment with Volaton G granulates (Phoxim), an insecticide formulated from terbufos with systemic and contact action for ground application and often used in greenhouses (Strajnar and Širca, 2011; Gerič Stare et al., 2017). Currently, chemical control of *M. luci* relies on pesticides containing fluopyram, a systemic, broad-spectrum active compound belonging to the benzamide chemical class and acts as a succinate dehydrogenase inhibitor fungicide and nematicide. Application of Velum^®^ Prime (Bayer) in infested greenhouses by *M. luci* has been proven effective in reducing nematode populations (Širca et al., 2024). Two studies evaluated the effectiveness of seed-applied fluopyram, a systemic nematicide, for controlling the RKN *M. incognita* in soybean and cotton production systems. It significantly reduced infection and root damage caused by this species (Spinks et al., 2000; Hawk and Faske, 2020). According to Grabau et al., 2021, the fumigant 1,3-dichloropropene provided the most consistent control of *M. incognita* and resulted in increased tomato yield while the non-fumigant nematicides (fluopyram and oxamyl) showed variable efficacy.

### 5.4 Bionematicides and biological control

Stricter European Union regulations on agricultural chemicals have increased interest in environmentally friendly approaches for nematode management in sustainable and organic farming systems. Bionematicides, based on microorganisms and their natural compounds, offer an effective and environmentally safe approach within integrated pest management (European Parliament, 2009: Stirling, 2011; Hildago-Diaz and Kerry, 2008).

In the study of Strajnar and Širca (2011), aqueous extract of *Tagetes erecta* and the commercial natural product azadirachtin (NeemAzal-T/S), extracted from the seeds of Indian Neem tree (*Azadirachta indica*), applied at 0.3% and 1% reduced *M. luci* (previously identified as ‘*M. ethiopica’;* Gerič Stare et al., 2017) reproduction by 2.6, 4.4 and 6.3 times compared to untreated controls. Additionally, deformed non-viable eggs of *M. luci* were observed after treatment with NeemAzal-T/S, while foliar application of Thiacloprid as Imidacloprid had no effect on nematode reproduction (Strajnar and Širca, 2011).

The effects of two natural compounds (juglone and 1,4-naphthoquinone), extracted from walnut husk residues, on *M. luci* life cycle showed that both compounds increased the *in vitro* mortality of J2 and reduced hatching by 50%. In pot assays with tomato cv. Coração-de-Boi, J2 infection was also reduced by 80%. Therefore, walnut residues can be valorized as renewable sources of juglone/1,4-naphthoquinone-based products and potentially employed as bionematicides against *M. luci* (Maleita et al., 2022b). The transcriptome profile of *M. luci* J2 in response to 1,4-NTQ exposure was determined by RNA-seq. A high number of downregulated genes were found between 1,4-naphthoquinone treatment and water control, reflecting the inhibitory effect of this compound on *M. luci*, with a great impact on processes related to translation (ribosome pathway) (Cardoso et al., 2023).

Many synthetic nematicides have been replaced by bacterial secondary metabolites (avermectins), and biological agents such as the fungi *Pochonia chlamydosporium*, *Myrothecium verrucaria*, *Purpureocillium lilacinus, Trichoderma* spp., and *Metarhizium* spp., and bacteria of the genera *Pasteuria*, and *Bacillus* (Migunova and Sasanelli, 2021; Pires et al., 2022). Rhizobacteria *Bacillus firmus* (Bacillales: Bacillaceae) is among the most frequently used control agent (Tian et al., 2007). Assessment of nematicidal activity of *B. firmus* strains *in vitro* showed significant reduction in number of hatched and motile J2, while in pot experiments *B. firmus* significantly reduced Rf of *M. luci* compared to the untreated control (Susič et al., 2019). Following this research work, nematicidal activity of *B. firmus* I-1582 against *M. luci* was evaluated, in pot and microplot experiments, and a reduction of nematode population by 51% and 53% compared to the untreated control was reported (Susič et al., 2020b). Hyperspectral imaging in the 400–2500 nm spectral range also successfully differentiated *B. firmus*-treated tomato plants and control plants, with 97.4% and 96.3% accuracy in pot and microplot experiments, respectively, showing the ability of this technology to rapidly assess the success of biological measures for RKN control (Susič et al., 2020b).

Overall, biological control represents a promising and environmentally sustainable tool for the management of *M. luci* within integrated pest management systems. Although numerous studies have demonstrated the efficacy of plant-derived compounds and microbial agents, further research is required to better understand their mechanisms of action, optimize their field performance, and ensure consistent efficacy under diverse agroecological conditions. Although research on other *Meloidogyne* species suggest significant effects on rhizosphere microbial communities and biological control efficiency, interactions between *M. luci* and the soil microbiome are still insufficiently examined and it is crucial to be also studied (Wolfgang et al., 2019; Kudjordjie et al., 2024). Continued advances in this field are expected to support the development of reliable biocontrol strategies for future nematode management.

## 6 Pest risk analysis

The pest risk assessment included the assessment of probability of introduction in a new area, host plants, pathways of spreading and potential economic impact (EPPO, 2023b). The probability of introduction is based on RKN geographical distribution (South America, Asia and Europe); and host plants refers to significance of the disease for economically important plants and host suitability (related to the reproduction factor, Rf). According to Sasser et al., 1984, Rf represents the ratio between final nematode population density (Pf) and initial nematode population density (Pi). Plants with Rf ≥ 1 were classified as good hosts; with 0.1 < RF < 1.0 as poor hosts; and RF ≤ 0.1 as non-hosts (Sasser et al., 1984).

Like other RKN, *M. luci* nematodes move only a few meters annually in the soil, but they can be disseminated to different regions through human activities, transport of infected plants and infested soil, soil adhering to farm implements and water irrigation. In Chile, it is suspected that the transport of grapevine nursery stock has probably resulted in infestations in vineyards. For this reason, national and international quarantine measures are being established to decrease the risk of spread and introduction of new species into regions where they do not exist (EPPO, 2017).

Although the pathway of *M. luci* introduction remains unknown, if we consider a potential tropical origin of the species, probably, the pathways for their introduction in the EPPO region could have involve the transport of plants for planting with infected roots, bulbs, rhizomes, tubers and corms, and/or infested soil associated with underground host plant parts or infested growing soil. These and soil adhering to farm implements and water irrigation are possible pathways that have been contributing to the dissemination of *M. luci* throughout the EPPO region (Širca et al., 2004; Gerič Stare et al., 2018; Conceição et al., 2012; Maleita et al., 2012; Aydınlı et al., 2013; Aydınlı and Mennan, 2016; Aydınlı, 2018; Maleita et al., 2018; Santos et al., 2019; Susič et al., 2020a; Rusinque et al., 2021; Aydınlı and Mennan, 2022; Bačić et al., 2023, 2025; **Table 1**). Therefore, the import of soil and below-ground plant parts have been already regulated in several EPPO countries (EU Regulation, 2016, 2019) and the use and transport of clean, healthy, nematode-free planting material is mandatory to limit the spread of RKN.

According to EPPO report of pest risk analysis (EPPO, 2023b) and the current distribution of *M. luci* and impact on Slovenia and Türkiye, the magnitude of impact was evaluated as high, due to expected losses in the fields and greenhouses (Gerič Stare et al., 2018; Maleita et al., 2018; Aydınlı et al., 2019; Şen and Aydınlı, 2021; Aydınlı and Mennan, 2022; Maleita et al., 2022a). Nevertheless, due to the lack of available data on *M. luci* economic damage, misidentification, cross-reaction with *M. incognita* and difficulties in epidemiological assessment, it was estimated that this classification was associated with a moderate degree of uncertainty.

## 7 Conclusion and perspectives

In this review, we highlighted the novel insights by focusing on several studies in identification, bio-ecology and control of *M. luci* using conventional and new technologies. This destructive nematode threatens agriculture worldwide, especially in Europe hence its quarantine status in the EPPO region. A diagnostic EPPO protocol for this emerging pest is currently under development. Climate change has resulted in the migration of previously undocumented tropical RKN species such as *M. luci* into new field environments across the world. Substantial research efforts have been dedicated to the accurate identification of *M. luci*, which represents a critical first step toward implementation of effective control strategies. The control strategies of this polyphagous species based on cultural measures, such as crop rotations, could be ineffective against this pathogen. Traditional nematicides have been banned because of adverse effects on the environment and human health. Recently, researchers have mainly concentrated on plant host resistance and biological control. To manage this emerging pathogen, substantial investments are required to lead research on new *M. luci* diagnosis methods, assessing the distribution of *M. luci* by specific surveys, pathogen virulence, genetic diversity, population genetic structure, evolution, parasitism mechanisms and *M. luci*-soil microbiome interactions. These measures would include restrictions on plant material movement and interruption of susceptible host varieties until nematode densities fall below the detection threshold.
