# Supplementary material for: Meloidogyne luci: an emerging threat to agriculture and challenges in diagnostics and sustainable management
Source: Front Plant Sci. 2026 Jul 7;17:1867596. doi: 10.3389/fpls.2026.1867596 (PMC13385228; doi:10.3389/fpls.2026.1867596)
Supplement: Supplementary file 1 [file Table1.docx]

**Supplementary material**

**Table S1.** The amplification conditions for identification of *Meloidogyne luci* with primers Mlf/Mlr. (Maleita et al., 2021)

| Steps | Temperatures | Time | Cycles |
| --- | --- | --- | --- |
| Initial denaturation | 94℃ | 4 min | 1 |
| Denaturation | 94℃ | 30 s | 35 |
| Annealing | 70℃ | 45 s | 35 |
| Extension | 72℃ | 45 s | 35 |
| Final extension | 72℃ | 10 min | 1 |
| Hold | 4℃ |  |  |

**Table S2.** The amplification conditions for identification of *Meloidogyne ethiopica* group with primers Me309/Me549R (Gerič Stare et al., 2019).

| Steps | Temperatures | Time | Cycles |
| --- | --- | --- | --- |
| Initial denaturation | 94℃ | 5 min | 1 |
| Denaturation | 94℃ | 30 s | 35 |
| Annealing | 50℃ | 30 s | 35 |
| Extension | 72℃ | 30 s | 35 |
| Final extension | 72℃ | 5 min | 1 |
| Hold | 4℃ |  |  |

**Table S3.** The amplification conditions for identification of tropical root-knot nematodes group with primers C2F3/Mt575 (Gerič Stare et al., 2019).

| Steps | Temperatures | Time | Cycles |
| --- | --- | --- | --- |
| Initial denaturation | 94℃ | 5 min | 1 |
| Denaturation | 94℃ | 30 s | 35 |
| Annealing | 54℃ | 30 s | 35 |
| Extension | 72℃ | 1 min | 35 |
| Final extension | 72℃ | 5 min | 1 |
